# Intention to Seek Care for Symptoms Associated With Gynecologic Cancers, HealthStyles Survey, 2008

**Published:** 2011-10-15

**Authors:** Katrina F. Trivers, Juan L. Rodriguez, Nikki A. Hawkins, Lindsey Polonec, Cynthia A. Gelb, Crystale Purvis Cooper

**Affiliations:** Division of Cancer Prevention and Control, Centers for Disease Control and Prevention; Centers for Disease Control and Prevention, Atlanta, Georgia; Centers for Disease Control and Prevention, Atlanta, Georgia; Centers for Disease Control and Prevention, Atlanta, Georgia; Centers for Disease Control and Prevention, Atlanta, Georgia; Soltera Center for Cancer Prevention and Control, Tucson, Arizona

## Abstract

**Introduction:**

Women with ovarian cancer typically experience symptoms before diagnosis; such symptoms for other gynecologic cancers have not been systematically studied. We investigated which symptoms of gynecologic cancers prompt intention to seek care among women and whether demographic differences in intention exist. This study was undertaken, in part, to inform development of the Centers for Disease Control and Prevention's campaign, Inside Knowledge: Get the Facts About Gynecologic Cancer.

**Methods:**

We analyzed the 2008 HealthStyles dataset (n = 2,991 women), an annual, cross-sectional, national mail survey. We calculated weighted percentages of women who indicated an intention to seek care for symptoms (defined as intention to call or see a doctor) by demographic characteristics and level of concern about developing a gynecologic cancer. We evaluated independent predictors of intention to seek care for each symptom.

**Results:**

For most symptoms, more than 50% of women reported an intention to seek care. Greater percentages of women indicated an intention to seek care for symptoms clearly gynecologic (eg, 91%, postmenopausal bleeding) than for symptoms not clearly gynecologic (eg, 37%, feeling full after eating a small amount). For most symptoms, after adjustment, black women, postmenopausal women, and women with greater concern about developing gynecologic cancers were more likely than their counterparts to intend to seek care.

**Conclusion:**

Intention to seek care differed by race, menopausal status, and level of concern about developing a gynecologic cancer. These findings will help in developing messages to educate women about the array of gynecologic and nongynecologic cancer symptoms.

## Introduction

Gynecologic cancers (cervical, ovarian, uterine, vaginal, and vulvar) are diagnosed in approximately 80,000 women per year (1); with the exception of cervical cancer, no screening tests are available to decrease morbidity or mortality from these cancers. Promoting awareness of gynecologic cancer symptoms may be a strategy for earlier detection of at least some gynecologic cancers (2). Symptoms noted by patients before diagnosis of gynecologic cancers have not been well studied. Most research on gynecologic cancer symptoms has focused on ovarian cancer. Women with ovarian cancer typically experience some symptoms before diagnosis, and they experience them more frequently than women without cancer, but symptoms are often nonspecific and are also present in many women without cancer (3-6). Common symptoms of ovarian cancer include abdominal distention, bloating, and unusual abdominal pain (3-12), each of which may not be immediately recognizable as a symptom of ovarian or other gynecologic cancers (13).

Gynecologic cancers have diverse symptoms. Symptoms specific to each of the 5 main types of gynecologic cancers may include, but are not limited to, the following:

- Cervical cancer: unusual vaginal bleeding (eg, irregular periods, bleeding after sexual intercourse) or vaginal discharge (14,15).
- Ovarian cancer: having pelvic or abdominal pain or pressure, back pain, or bloating; having to urinate more frequently or urgently than usual; feeling full after eating a small amount of food; having unusual vaginal discharge; and postmenopausal vaginal bleeding (3-6,8,10,12-14,16,17).
- Uterine cancer: postmenopausal vaginal bleeding, bleeding between periods, periods that are either longer or heavier than normal, and pelvic or abdominal pain (14,18).
- Vaginal cancer: having unusual vaginal bleeding, especially after sexual intercourse, or having to urinate more frequently or urgently than usual (14,19).
- Vulvar cancer: persistent itching of the vulva, changes in the color of the vulva, so that it looks redder or whiter than usual; rash- or wart-like skin changes in the vulva; and a persistent sore on the vulva (14,20).

These symptoms may also be linked to various health conditions unrelated to gynecologic cancers (3-6).

The Centers for Disease Control and Prevention (CDC), in collaboration with the Department of Health and Human Services' Office on Women's Health, established the Inside Knowledge: Get the Facts About Gynecologic Cancer campaign to support the Gynecologic Cancer Education and Awareness Act of 2005, or Johanna's Law, which was signed into law on January 12, 2007 (www.cdc.gov/cancer/gynecologic/pdf/JohannasLaw.pdf). The campaign is charged with educating women and health care providers about the signs and symptoms, screening tests (if available), prevention strategies, and risk factors associated with the 5 main types of gynecologic cancer. Furthermore, the campaign encourages women to pay attention to what is normal for them, so they may recognize any persistent changes and seek care in an appropriate and timely manner.

Data on women's intentions to seek care are needed to help shape education and awareness messages of the Inside Knowledge campaign and to provide a baseline evaluation of existing campaign messages. The objective of this study was to investigate symptoms of gynecologic cancers that prompt intention to seek care among women and whether demographic differences in intention to seek care exist.

## Methods

We used secondary data from the 2008 HealthStyles survey, to examine the relationship between intention to seek care and demographic characteristics. Analyses of HealthStyles, a de-identified dataset, were declared exempt by CDC's institutional review board.

### Study design and sample population

HealthStyles is an annual, cross-sectional, national mail survey of adults aged 18 years or older conducted by Porter Novelli, a global public relations agency. The survey includes questions about health beliefs, attitudes, and behaviors and is mailed to people enrolled in the Synovate Global Opinions Panel (21). Porter Novelli develops the survey annually on the basis of current health topics and recommendations from public health agencies. Results from HealthStyles data items are similar to results from the Behavioral Risk Factor Surveillance System (22). In 2008, CDC's Division of Cancer Prevention and Control licensed items from HealthStyles. The 2008 HealthStyles survey was mailed to 7,000 of 10,108 people who had returned an earlier related survey, ConsumerStyles; people were selected to participate in the earlier survey through stratified random sampling to create a nationally representative sample. Participants in ConsumerStyles receive small incentives for participation (a 30-min telephone calling card or a lottery entry to win between $50 and $1,000). We used a supplement to ensure adequate representation of low-income and racial/ethnic minority groups. HealthStyles surveys were returned by 5,399 participants (77.1%[5,399 of 7,000]). We poststratified and weighted the data according to the US Census distribution of age, race/ethnicity, sex, household size, and annual household income.

### Outcome measures

Only women (n = 2,991) answered questions on gynecologic cancers and intention to seek care. We assessed intention through 2 sets of questions. The first set, comprising 10 questions, each referring to 1 symptom, asked women how they would respond to each symptom ("If you had any of the following, would you call or see a doctor?"). Nine of these symptoms are typically considered gynecologic (ie, clearly related to the reproductive system). Throughout this article, we refer to these 9 symptoms as "abnormal," although the survey questions did not refer to them as such. In this set of questions, we also included a question on intention to seek care for influenza-like symptoms (developed and included before the outbreak of H1N1), which are not typically associated with gynecologic cancers, as a control. The second set, comprising 5 questions, assessed intention to seek care for 5 chronic symptoms. The survey asked, "If it was not normal for you, and you experienced any of the following every day for 2 weeks or longer, would you call or see a doctor?" These 5 symptoms are typically considered nongynecologic (ie, not obviously related to the reproductive system). Throughout this article, we refer to these 5 symptoms as "abnormal and persistent" symptoms, although the survey did not refer to them as such. Response options for both sets of questions on intention to seek care were yes, no, or "not sure" for each symptom.

The survey also asked participants about their concern about developing each of the 5 gynecologic cancers; participants were asked to express concern on a scale of 1 (not at all concerned) to 4 (very concerned).

The survey collected data on age, annual household income, education, employment status, marital status, menopausal status, race/ethnicity, tobacco use, alcohol use, having a mammogram, and having a Papanicolaou (Pap) test. We defined menopausal status on the basis of the question, "Have you gone through menopause (end of menstruation)?" Possible responses to this question were yes, no, "I'm going through it now," and not sure.

### Statistical analyses

Our analyses were mostly descriptive; we tested no hypotheses. We calculated intention to seek care as the weighted percentage of women, for each symptom, who answered yes to the question on whether they would call or see a doctor. We calculated weighted percentages for demographic characteristics and concern about developing a gynecologic cancer. We established subcategories for each covariate. We categorized menopausal status into 3 groups: postmenopausal was established by the response "Yes, I've gone through menopause," perimenopausal by the response "I'm going through menopause now," and premenopausal by the response "No, I haven't gone through menopause." We calculated mean gynecologic cancer concern (mean concern, 2.4) by averaging the scores for each of the 5 cancers.

We performed separate analyses for each of the 15 symptoms. We entered each covariate (eg, age) into its own unadjusted logistic regression model; the outcome of interest was intention to seek care for each symptom. If the covariate was found to be significantly associated (*P* < .05) with intention to seek care for a majority of the symptoms, we included it in the full model for each symptom. The full model also included reported intention to seek care for influenza-like symptoms to control for level of generalized health care use and concern. The full model for abnormal symptoms differed from the full model for abnormal and persistent symptoms. From the adjusted models, we estimated a weighted predicted probability (expressed as a percentage) of intention to seek care for each covariate by setting the remainder of the covariates to their reference level. The predicted probability of intention to seek care for each referent group is therefore equivalent. We calculated weighted, adjusted percentages, rather than odds ratios, because we were more interested in estimating absolute levels of intention to seek care than relative levels. We excluded women with missing data on a variable from any analysis of that variable. We used SAS software, version 9.2 (SAS Institute, Inc, Cary, North Carolina) for all analyses. We did not use higher-order statistics (eg, tests for interaction) because our analysis did not test a hypothesis.

## Results

Most survey participants were white (65%), aged 45 years or older (65%), married (61%), had at least some college education (66%), and had an annual household income of $40,000 or more (54%). Overall, more than 50% of women reported an intention to seek care for most of the 15 symptoms (Table 1); only 41.0% reported intention to seek care for bloating and only 37.2% for feeling full after eating a small amount of food. A greater percentage of women reported an intention to seek care for gynecologic symptoms (eg, postmenopausal vaginal bleeding, 90.5%; vaginal itching, 90.4%; vaginal discharge, 83.1%) than for nongynecologic symptoms (eg, bloating, feeling full after eating a small amount of food). The exception to this finding was that, among premenopausal women, only 54.9% intended to seek care for periods that lasted longer than 7 days and only 57.6% intended to seek care for vaginal bleeding between periods, both of which are gynecologic symptoms.

In unadjusted analyses of abnormal symptoms, younger women (aged 18-34 y) were less likely than older women (aged ≥50 y), and premenopausal women were less likely than postmenopausal women, to report intention to seek care. Black women (and to a lesser degree, Hispanic women) were more likely than white women, and women with greater concern about developing a gynecologic cancer (≥2.4) were more likely than women with less concern (<2.4), to report intention to seek care for abnormal symptoms. We found no consistent differences in intentions to seek care for abnormal symptoms by annual household income, education, employment status, or marital status.

In unadjusted analyses of abnormal and persistent symptoms, younger women were less likely than older women, premenopausal women were less likely than postmenopausal women, and women with middle-level incomes ($25,000-$59,999) were less likely than women in the highest level of income (≥$60,000), to report intention to seek care. We found no consistent differences in intentions for abnormal and persistent symptoms by education, employment status, marital status, race/ethnicity, or concern about developing a gynecologic cancer.

Results were largely unchanged after adjustment, except the effect of age, which became statistically nonsignificant for many symptoms. After adjustment, black and Hispanic women (compared with white women), women who reported they would seek care for influenza-like symptoms (compared with women who would not), postmenopausal women (compared with premenopausal women), and women with greater concern about gynecologic cancer (compared with women less concerned) were more likely to report they would seek care for abnormal symptoms (Table 2). For abnormal and persistent symptoms, women with annual incomes of $60,000 or more, women who reported they would seek care for influenza-like symptoms, and postmenopausal women were more likely to report intention to seek care than their counterparts, after adjustment (Table 3). We found none of the following health-related behaviors to be a confounder: tobacco use, alcohol use, having a mammogram, or having a Pap test.

## Discussion

Women's intention to seek care differed by age and demographic characteristics. Postmenopausal women, black women, women more concerned about developing a gynecologic cancer, and women with annual incomes of $60,000 or more were more likely than their counterparts to report they would seek care for most symptoms. Why black women, and to a lesser degree, Hispanic women, are more likely to report intention to seek care for abnormal symptoms is not entirely understood and deserves further study. Women were generally more likely to report intention to seek care for gynecologic symptoms than nongynecologic symptoms; the exceptions were for premenopausal women, who reported low levels of intention to seek care (55% and 58%) for 2 gynecologic symptoms. These data suggest a need to educate women about the array of gynecologic cancer signs, symptoms, and risk factors, a fundamental aim of CDC's Inside Knowledge campaign and other public health initiatives.

Although we do not have data on reasons for women's intentions to seek care for certain symptoms, a possible interpretation is that women experience some gynecologic symptoms relatively infrequently, and thus, they consider them to be more serious than nongynecologic symptoms (eg, gastrointestinal symptoms). One study showed that gastrointestinal symptoms were not recognized as serious by women (23) despite their presence before a diagnosis of ovarian cancer (3-6,8-12). This lack of recognition is potentially problematic; in 1 study, women who visited a physician and presented with gastrointestinal symptoms were more likely to be diagnosed with late-stage ovarian cancer and received surgical evaluation less frequently than women who presented with gynecologic symptoms (12).

Whether earlier recognition of symptoms will translate into earlier detection and improved outcomes for women with gynecologic cancers is unknown. The predictive value of symptoms for early detection of ovarian cancer has been estimated to be low, and therefore it may have limited clinical use (24).

Although studies have estimated women's recognition of ovarian cancer symptoms at 26% to 47% (13), we did not assess whether participants associated the symptoms included in our study with gynecologic cancer. Women in our study were asked to describe their intention to seek care related to symptoms that they may or may not have recognized as being associated with gynecologic cancers. Our focus on care seeking was not intended to reflect women's knowledge about symptoms' link to ovarian or other gynecologic cancers but instead identifies gynecologic cancer symptoms that are most likely to prompt respondents to seek health care. This focus is consistent with the Theory of Planned Behavior (25) in which behavioral intentions, or a person's perceived likelihood of performing a certain behavior, have been found to be a strong predictor of actual behavior.

This study has limitations. We asked about intentions to seek care, which may not be equivalent to actual care-seeking behavior. Because our data are self-reported (and subject to response bias, including social desirability bias) and reflect only intention to seek care, they may overestimate actual care-seeking behavior. Our survey was not population based, although it was weighted to be representative of the US population. Also, data on additional possible confounders, such as access to medical care, health insurance, and additional psychosocial constructs (eg, risk perception, worry) were not available. However, the survey had a high response rate and a large sample size, enabling us to consider many characteristics potentially associated with intention to seek care.

Future studies should explore why women choose to seek care for certain symptoms (eg, do they consider some symptoms more worrisome than others?) and investigate other factors that may influence actual care seeking. The identification of diseases or conditions women associate with these symptoms would help in the design of educational materials and intervention efforts.

To our knowledge, this is one of only a few studies to investigate ovarian cancer symptoms that prompt an intention to seek care among US women and the first to investigate symptoms of other gynecologic cancers. The results of our study add to the growing body of literature on whether certain symptoms prompt an intention to seek care among certain groups of women. CDC's Inside Knowledge campaign and other public health initiatives should develop communication and education strategies to increase awareness of gynecologic cancer symptoms, with a focus on symptoms that may manifest as nongynecologic problems. Efforts should encourage appropriate care seeking among all groups of women but pay particular attention to women who may be less likely to seek care, such as white women, premenopausal women, and women with low incomes.

## Figures and Tables

**Table 1:** Responses of Women (N = 2,991), Study on Intention to Seek Care for Symptoms of Gynecologic Cancers, HealthStyles Survey, 2008

Symptom	**Response, %**		

	**Yes**	**No**	**Not Sure**
**Abnormal, predominately gynecologic^a^**			
Rash or sore on your genitals that does not go away	91.8	5.6	2.6
Vaginal bleeding after you have gone through menopause (postmenopausal women only)	90.5	6.3	3.2
Vaginal itching that does not get better with over-the-counter treatments/creams	90.4	5.7	3.8
Vaginal discharge that is not normal for you	83.1	10.7	6.2
Skin on your genitals becoming redder or whiter than is normal for you	82.1	9.1	8.9
Vaginal bleeding (not related to your period) after sex on 2 or more occasions	77.1	13.4	9.6
Pelvic or abdominal pain after sex	63.2	20.5	16.3
Influenza-like symptoms (eg, fever and body aches) for more than 3 days^b^	62.7	25.2	12.1
Vaginal bleeding between periods (premenopausal women only)	57.6	32.1	10.3
Period that lasts more than 7 days (premenopausal women only)	54.9	30.9	14.2
**Abnormal and persistent, predominately nongynecologic^c^**			
Pelvic or abdominal pain	88.9	6.4	4.7
Urinating more often or more urgently than usual	72.7	18.0	9.4
Back pain	59.0	27.6	13.4
Bloating	41.0	40.5	18.5
Feeling full after eating a small amount of food	37.2	41.5	21.3

a Survey participants were asked, "If you had any of the following, would you call or see a doctor?" Except for the question on influenza-like symptoms, these symptoms are typically considered gynecologic (ie, clearly related to the reproductive system) and are referred to as abnormal in this table (but were not during the survey).

b We included a question on intention to seek care for influenza-like symptoms, which are not typically associated with gynecologic cancers, as a control.

c Survey participants were asked, "If it was not normal for you and you experienced any of the following every day for 2 weeks or longer, would you call or see a doctor?" These symptoms are typically considered nongynecologic (ie, not obviously related to the reproductive system) and are referred to as abnormal and persistent in this table (but not during the survey).

**Table 2.** Model-Adjusted Predicted Probabilities (%) of Intention to Seek Care for Abnormal, Predominately Gynecologic Symptoms, by Demographic Characteristics, Among Women (N = 2,991), HealthStyles Survey, 2008^a^

**Characteristic**	**Genital Rash or Sore**	**Vaginal Bleeding After Menopause^b^**	**Vaginal Itching**	**Vaginal Discharge**	**Skin on Genitals Is Redder/Whiter**	**Vaginal Bleeding After Sex^c^**	**Pelvic or Abdominal Pain After Sex**	**Vaginal Bleeding Between Periods^d^**	**Period That Lasts >7 Days^d^**
**Age, y**									
18-34	83.7	NC	77.7	57.9^f^	58.2	53.4	24.3	40.1	32.5
35-49	80.9	60.6^f^	79.7	61.5^f^	58.5	53.5	31.4	39.5	37.3
≥50 (Reference)	80.9	81.9	77.2	74.4	61.7	60.3	31.3	42.3	33.6
Race									
Black	84.0	93.5	87.5^f^	84.7^f^	80.8^f^	76.5^f^	48.5^f^	68.5^f^	50.2^f^
Hispanic	85.8	82.2	83.2	80.3^f^	72.3^f^	67.2	31.6	51.9^f^	40.7^f^
Other	69.3^f^	89.1	66.8^f^	78.6	58.0	64.8	33.0	41.1	28.7
White (Reference)	80.9	81.9	77.2	74.4	61.7	60.3	31.3	42.3	33.6
**Would seek care for influenza-like symptoms**									
Yes	96.3^f^	93.9^f^	92.7^f^	90.9^f^	86.5^f^	79.5^f^	67.1^f^	54.3^f^	53.5^f^
No/not sure (Reference)	80.9	81.9	77.2	74.4	61.7	60.3	31.3	42.3	33.6
**Menopausal status**									
Post	83.4	NA	83.5	79.0	67.9	68.9^f^	41.6^f^	NA	NA
Peri	83.8	NA	83.9	77.9	68.1	70.1^f^	42.2^f^	NA	NA
Pre (Reference)	80.9	NA	77.2	74.4	61.7	60.3	31.3	NA	NA
**Concern about developing a gynecologic cancer^e^**									
<Mean (Reference)	80.9	81.9	77.2	74.4	61.7	60.3	31.3	42.3	33.6
≥Mean	83.8	88.8^f^	81.3	78.2	70.0^f^	68.6^f^	45.7^f^	50.0^f^	40.3^f^

Abbreviations: NC, not calculated; NA, not applicable.

a Predicted probabilities are estimated from a logistic regression model including all variables listed in the table and are calculated by setting all the other variables to their reference level. Survey participants were asked, "If you had any of the following, would you call or see a doctor?" These symptoms are typically considered gynecologic (ie, clearly related to the reproductive system) and are referred to as abnormal in this table (but were not during the survey).

b Postmenopausal women only.

c On 2 or more occasions.

d Premenopausal women only.

e Participants responded on a scale from 1 (not at all concerned) to 4 (very concerned); the mean score was 2.4.

f Significantly different from reference group (*P* < .05).

**Table 3.** Model-Adjusted Predicted Probabilities (%) of Intention to Seek Care for Abnormal and Persistent, Predominately Nongynecologic Symptoms, by Demographic Characteristics, Among Women (N = 2,991), HealthStyles Survey, 2008^a^

	**Pelvic or Abdominal pain**	**Having to Urinate More Often**	**Back Pain**	**Bloating**	**Feeling Full After Eating a Small Amount**
**Age**					
18-34	87.0^b^	61.7	38.3^b^	24.3	18.6
35-49	82.1^b^	57.9	43.3	23.2	22.2
≥50 (Reference)	73.5	60.4	49.3	23.8	20.9
**Annual income, $**					
<25,000	59.7^b^	55.7	47.2	21.9	19.4
25,000-39,999	64.1^b^	52.0^b^	43.0^b^	16.9^b^	15.2^b^
40,000-59,999	67.9	55.0	43.7	19.9	17.9
≥60,000 (Reference)	73.5	60.4	49.3	23.8	20.9
**Would seek care for influenza-like symptoms**					
Yes	91.2^b^	78.9^b^	77.1^b^	48.5^b^	43.6^b^
No/not sure (Reference)	73.5	60.4	49.3	23.8	20.9
**Menopausal status**					
Post	85.9^b^	72.7^b^	46.1	34.2^b^	32.8^b^
Peri	83.0^b^	67.3	46.8	29.9	26.6
Pre (Reference)	73.5	60.4	49.3	23.8	20.9

a Predicted probabilities are estimated from a logistic regression model including all variables listed in the table and are calculated by setting all the other variables to their reference level. Survey participants were asked, "If it was not normal for you, and you experienced any of the following every day for 2 weeks or longer, would you call or see a doctor?" These symptoms are typically considered nongynecologic (ie, not obviously related to the reproductive system) and are referred to as abnormal and persistent in this table (but were not during the survey).

b Significantly different from reference group (*P* < .05).
